# Dosiomics-Based Prediction of Radiation-Induced Valvulopathy after Childhood Cancer

**DOI:** 10.3390/cancers15123107

**Published:** 2023-06-08

**Authors:** Stefania Chounta, Rodrigue Allodji, Maria Vakalopoulou, Mahmoud Bentriou, Duyen Thi Do, Florent De Vathaire, Ibrahima Diallo, Brice Fresneau, Thibaud Charrier, Vincent Zossou, Stergios Christodoulidis, Sarah Lemler, Veronique Letort Le Chevalier

**Affiliations:** 1 Université Paris-Saclay, Univ. Paris-Sud, UVSQ, CESP, Cancer and Radiation Team, F-94805 Villejuif, France; 2 INSERM, CESP, Cancer and Radiation Team, F-94805 Villejuif, France; 3 Gustave Roussy, Department of Clinical Research, Cancer and Radiation Team, F-94805 Villejuif, France; 4 Université Paris-Saclay, CentraleSupélec, Mathématiques et Informatique pour la Complexité et les Systèmes, F-91190 Gif-sur-Yvette, France; 5 Polytechnic School of Abomey-Calavi (EPAC), University of Abomey-Calavi, 01, Cotonou P.O. Box 2009, Benin; 6 Department of Radiation Oncology, Gustave Roussy, F-94800 Villejuif, France; 7 Gustave Roussy, Inserm, Radiothérapie Moléculaire et Innovation Thérapeutique, Paris-Saclay University, F-94800 Villejuif, France; 8 Gustave Roussy, Université Paris-Saclay, Department of Pediatric Oncology, F-94805 Villejuif, France; 9 Institut Curie, PSL Research University, INSERM, U900, F-92210 Saint Cloud, France; 10 Institut de Formation et de Recherche en Informatique, (IFRI-UAC), Cotonou P.O. Box 2009, Benin

**Keywords:** dosiomics, late effects, childhood cancer, dosimetry, radiotherapy, valvulopathy, random forest, imbalanced classification

## Abstract

**Simple Summary:**

Childhood cancer survivors are often prone to experiencing late effects due to treatment complications. Valvular Heart Disease is a known iatrogenic effect of radiation leakage to the heart during radiotherapy and is often linked with the occurrence of other cardiac diseases like heart failure. Early identification and treatment of survivors prone to develop valvular heart disease is an important public health issue that remains challenging. In the FCCSS, a voxel-scaled reconstruction of radiation dose to the heart is available for patients that had been treated with radiotherapy. This type of uncommon data allows us to take into consideration information on the dose level that was absorbed by the cardiac tissues, as well as on the spatial characteristics of radiation dose distribution to the heart. With the help of machine learning algorithms, we attempted to train models capable of accurately predicting survivors high at risk of experiencing a late valvular heart disease after radiotherapy for childhood cancer. We suggest that there is an underlying association of the radiation dose with the occurrence of a valvular heart disease that goes beyond the mean dose to the heart and can be explained by the combination of spatial and descriptive features of the dose.

**Abstract:**

Valvular Heart Disease (VHD) is a known late complication of radiotherapy for childhood cancer (CC), and identifying high-risk survivors correctly remains a challenge. This paper focuses on the distribution of the radiation dose absorbed by heart tissues. We propose that a dosiomics signature could provide insight into the spatial characteristics of the heart dose associated with a VHD, beyond the already-established risk induced by high doses. We analyzed data from the 7670 survivors of the French Childhood Cancer Survivors’ Study (FCCSS), 3902 of whom were treated with radiotherapy. In all, 63 (1.6%) survivors that had been treated with radiotherapy experienced a VHD, and 57 of them had heterogeneous heart doses. From the heart–dose distribution of each survivor, we extracted 93 first-order and spatial dosiomics features. We trained random forest algorithms adapted for imbalanced classification and evaluated their predictive performance compared to the performance of standard mean heart dose (MHD)-based models. Sensitivity analyses were also conducted for sub-populations of survivors with spatially heterogeneous heart doses. Our results suggest that MHD and dosiomics-based models performed equally well globally in our cohort and that, when considering the sub-population having received a spatially heterogeneous dose distribution, the predictive capability of the models is significantly improved by the use of the dosiomics features. If these findings are further validated, the dosiomics signature may be incorporated into machine learning algorithms for radiation-induced VHD risk assessment and, in turn, into the personalized refinement of follow-up guidelines.

## 1. Introduction

Childhood cancer (CC) survival rates have risen over the past decades in high-income countries, owing to advances in oncology treatment [1,2,3]. Radiotherapy, in particular, radically improves cancer survival in many cases [4], and modern optimizations [5,6,7,8] have had a substantial impact in reducing toxicity and side risks. Meanwhile, during treatment with radiotherapy, healthy tissues cannot be avoided entirely; this can potentially lead childhood cancer survivors to suffer chronic damage; especially at risk are those who did not benefit from modern protocols.

Identifying high-risk individuals and providing them with early diagnosis and treatment is an ever-present public health concern, especially with such vulnerable populations as CC survivors. While data-driven clinical predictions are an ancient medical practice, modern machine learning algorithms can significantly improve accuracy and become a helpful asset in predicting the late cardiac effects of CC treatments [9,10].

According to the American Childhood Cancer Survivors Study, two out of three survivors experience at least one late iatrogenic effect [11]. Heart disease is among the known complications of CC treatment [12,13,14,15]. In this study, we are interested in identifying CC survivors with an increased risk of experiencing severe Valvular Heart Disease (VHD) several decades after treatment for CC.

It has been established that the risk of experiencing VHD increases with the level of radiation absorbed by heart tissues during radiotherapy [16,17,18]. In addition, an association of high (>25 Gy) radiation doses to the heart with the occurrence of VHD has already been reported, both for adult [19,20] and pediatric [16,21,22] cancer treatment. There is, however, an open question concerning the potential risk induced by extensive low and moderate radiation doses to the cardiac region. In [23], the relative risk of cardiac events was expressed with respect to the percentage of the heart volume which absorbed a dose between 5 and 20 Gy, and was found to be significant when more than 50% of the heart volume was affected. Meanwhile, in [16], it was suggested that a cut-off might exist below which there is no risk of subsequent Valvular Heart Disease. In [18], evidence was provided that such a threshold could be around 5 Gy, and that doses between 5 and 20 Gy absorbed by more than 90% of the heart volume are statistically associated with the occurrence of VHD. Consequently, we hypothesize that some distribution patterns could also be associated with the occurrence of VHD.

The most common explanatory variables to model the radiation-induced risk of VHD are the mean or the median dose to the heart [17,19,24]. However, mean and median dose to the heart do not provide insight into the role of spatial heterogeneity of received doses; more specifically, they do not allow an exhaustive representation of the characteristics of the dose distribution when it is heterogeneous. This issue remains understudied in the literature, mainly due to a lack of adequate whole-body voxel-scale data. In some studies with access to such data, the role of dose–volume histogram parameters in experiencing a cardiac disease has been investigated with fruitful results [18,19,23,25]. These first results encourage further investigation of the potential role of heart dose heterogeneity in experiencing VHD, using more systematic approaches.

In this study, we adopted the dosiomics approach, which involves extracting first-order statistics and 3D spatial features from radiation dose distribution, to go one step further. Studies have been exploring the role of dosiomics in risk modeling to predict radiation-induced temporal lobe injury [26], radiation pneumonitis [27], locoregional recurrences after treatment for head and neck carcinoma [28], and radiation-induced hypothyroidism [29], to name a few applications. Dosiomics features have proven promising and, in some cases, more effective than the conventionally used dose–volume histogram parameters [29,30]. To our knowledge, this is the first study where dosiomics are extracted from the heart dose to estimate the risk of subsequent VHD. We chose to tackle the subject as a classification problem of VHD prediction several decades after treatment with radiotherapy for CC. We grew Random Forests based on the mean heart dose (MHD) (baseline model) and dosiomics features of survivors that experienced VHD, to deduce a signature in high-risk survivors. The main objectives of this study were to identify critical variables in risk estimation (*dosiomics signature*) and to grow efficient Random Forests that can accurately screen high-risk CC survivors prone to experiencing VHD.

## 2. Materials and Methods

### 2.1. Population and Identification of VHD Events

In the FCCSS cohort, information on demographic and clinical characteristics were gathered for 7670 5-year CC survivors treated between 1945 and 2001 for the most common childhood solid cancers (defined according to the third edition of the International Classification of Childhood Cancer-ICCC-319 [31]) in 5 different cancer centers in France before the age of 21, as previously reported [12,18,32,33,34]. Of these, 7488 had complete data and were included in the analyses. The FCCSS was approved by a regional committee on ethics and the French national agency regulating data protection (Commission Nationale Informatique et Liberté, agreements no. 902287 and no. 12038829). All patients, parents, or guardians have signed a written informed consent form under national research ethics requirements. The present analysis included 7488 5-year survivors (97.7% of the FCCSS cohort) with complete treatment data.

Vital status was obtained for all patients and causes of death from cépiDC (Center of epidemiology on medical causes of death) [35], coded according to the 9th and 10th versions of the International Classification of Diseases and confirmed by the French Registry of Death [31]. Clinical and epidemiological follow-up is being performed to identify the occurrence of iatrogenic effects from self-administered questionnaires, cohort linkage with the French Hospital Database and health insurance information system [36], and clinical follow-up for the patients of Gustave Roussy.

VHD events were identified, validated, and graded according to the Common Terminology Criteria for Adverse Events (CTCAE version 4.0322 [37]). We considered only severe VHD cases (grade ≥3), since there are concerns that non-severe cardiovascular disease is often self-declared and could cause a reporting bias in the data [38]. We identified 81 (≈1%) survivors who had either experienced severe VHD before any other cardiac disease or for whom VHD was among their three first causes of death. Severe VHD is hereafter called VHD.

### 2.2. Voxelised Dosimetric Data: Dosimetry Factors and Dosiomics Features

**Figure 1:**
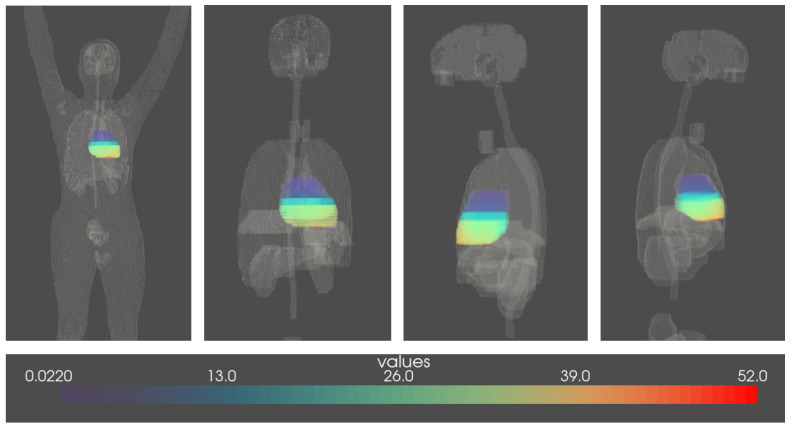
Representation of the voxelized heart–dose reconstruction; four views (front, back, left, and right) of one childhood cancer survivor; voxels are of size 2 mm^3^, and the color shades represent the level of the radiation dose (in Gy). This survivor was treated at 3.5 years old in 1961 for Hodgkin lymphoma and received a mean heart dose of 19.6 Gy.

Whole-body voxel-scale radiation dosimetry was available for 3902 patients who had received radiotherapy, following a methodology of absorbed dose reconstruction that has already been published [39,40]. For this study, we only included the heart dose reconstruction. An example is demonstrated in Figure 1.

The dosiomics definition is derived from the now well-established radiomics, a technique developed for image analysis [41,42], where voxel intensity plays the role of dose level. This allows high throughput extraction of numeric data (image ‘biomarkers’) from 3D images, in order to represent various aspects of the image characteristics (spatial patterns, texture, distribution statistics, etc.).

We extracted 93 dosiomics features from the dose to the heart using the pyradiomics package (3.0.1) [42]. The features can be categorized into six classes:

- Eighteen first-order statistics of the heart dose;
- Twenty-four Gray Level Co-occurrence Matrix (GLCM) features;
- Sixteen Gray Level Run Length Matrix (GLRLM) features;
- Sixteen Gray Level Size Zone Matrix (GLSZM) features;
- Fourteen Gray Level Dependence Matrix (GLDM) features;
- Five Neighboring Gray Tone Difference Matrix (NGLDM) features.

The complete list of features is provided in Appendix A (Table A1).

The extracted features provide information on the dose intensities and have already been described [42]. Shape features (2D and 3D) were not calculated, as they concern the size and shape of the region of interest. In the context of this study, the region of interest is the heart. As the shape and size of the organs have been approximated by phantoms for many survivors and there is often uncertainty in relation to organ contouring, it would not be informative to include size features in the models. The binwidth of dose histograms was set to 0.1 Gy where applicable (set according to the Freedman–Diaconis rule [43]).

### 2.3. Imbalanced Classification and Feature Selection

Our analyses concerned a retrospective cohort, and survivors experienced VHD up to 50 years after treatment for childhood cancer. We attempted to identify high-risk survivors with a supervised classification problem. However, only 1% of the survivors were diagnosed with severe VHD. Therefore, we were dealing with an imbalanced classification problem of identifying survivors diagnosed with severe VHD, where the prediction that no survivor was at risk would result in a 99% accuracy (Number of correct predictions/Total number of predictions).

Chen et al. [44] proposed two possible adaptations of the classic Random Forest algorithm to tackle the problem of imbalanced data: Weighted Random Forest (wtRF) and Balanced Random Forest (BRF). The wtRF is based on the idea of cost-sensitive learning to penalize misclassification of the minority class. A weight is assigned to each class and incorporated into two steps of the random forest algorithm: (i) in the tree induction procedure, class weights are used to weight the Gini criterion for finding splits, and (ii) in the terminal nodes of each tree, where class weights are again taken into consideration to determine the prediction according to a weighted majority vote. The BRF incorporates the idea of down-sampling the majority class during each bootstrap step by selecting a bootstrap sample from the minority class and then randomly drawing the same number of cases from the majority class.

To evaluate the models based on the extracted dosiomics features, we compared them to forests grown from the MHD. An adjusted version is also presented based on the following adjustment variables: biological sex, age (in years) and year of the first childhood cancer diagnosis, and chemotherapy exposure (binary: 1 if chemotherapy was administered during childhood cancer, 0 otherwise).

### 2.4. Modeling Workflow

Given the largely unbalanced nature of the dataset, particular attention was paid to avoiding biased estimates and overfitting. To increase the robustness of our results, we repeated our entire analysis pipeline over 30 random instances of train–test split. It should be noted that another strategy could have been cross-validation, but this has been shown to not provide better accuracy [45]. The 30 random and overlapping divisions of the training and test sets were chosen so as to respect the balance in relation to the proportion of VHD incidents.

**Figure 2:**
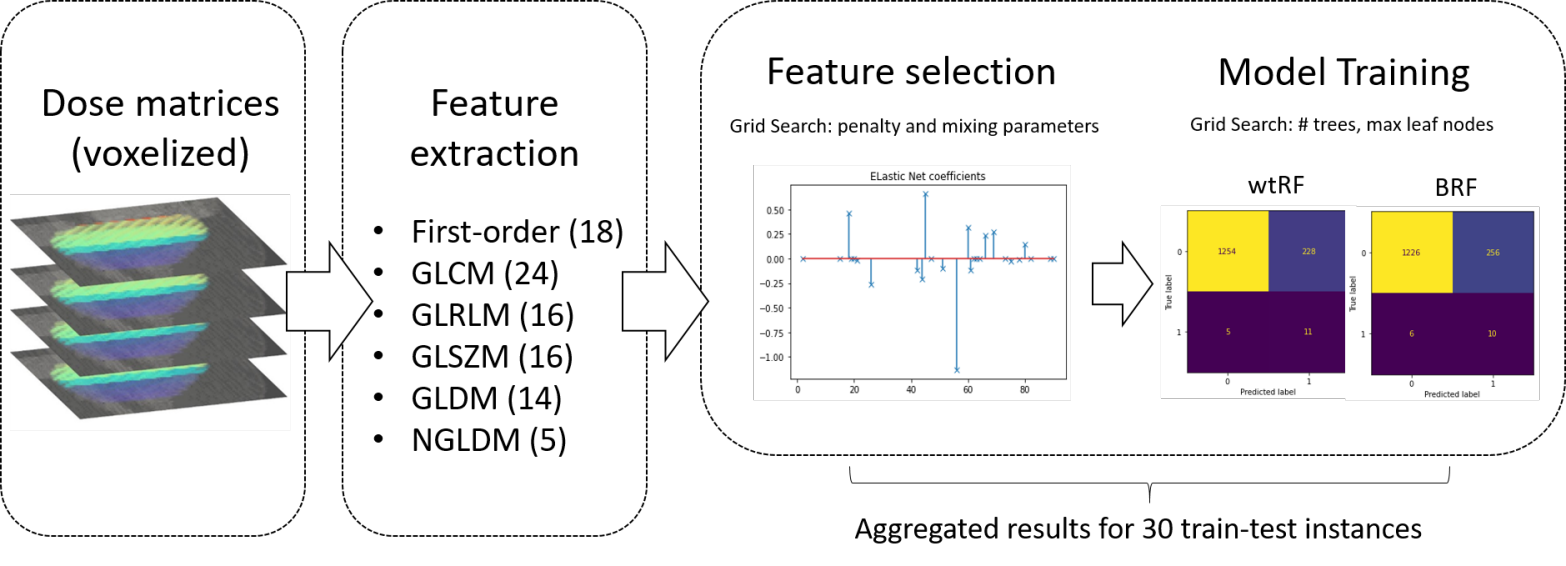
Workflow of the dosiomics-based models, as described in Section 2.4. We extracted 93 dosiomics features from the radiation dose to build the heart matrices, split the cohort into train–test groups 30 times, used the Elastic Net to do a variable selection, and after 5-fold cross validation for hyperparameters calibration (number (#) of trees and maximum leaf nodes) we, then, trained the weighted (wtRF) and balanced random forests (BRF). Then we calculated the metrics of performance for each of the two types of Random Forest by aggregating the results of the 30 splits.

For the dosiomics-based models, as illustrated in Figure 2, we started the pipeline with variable selection through an Elastic Net, which is appropriate when the variables form groups that contain highly correlated variables, as is the case with diosiomics [46]. The regularization hyper-parameters were tuned through a grid search with cross-validation. Then, we performed 5-fold cross-validation on the train set to calibrate the Random Forest parameters (number of trees to grow and maximum leaf nodes). We then calculated variable importances for each instance (computed as the mean and standard deviation of accumulation of the impurity decrease within each tree) and confusion matrices. From the confusion matrices, we calculated the following metrics, aggregated across the 30 instances: Sensitivity (Recall), Specificity, Balanced Accuracy (BA), and AUC ROC (defined below). Metrics results are presented in the corresponding section as average ± standard deviation. All *p*-values computed for the performance comparisons were obtained from t-tests under the assumption of variance homogeneity. For the MHD-based models, the pipeline was similar, except for the feature selection step.

### 2.5. Dosiomics Signature

Each presented feature was selected from at least 25 of the 30 iterations of the Elastic Net. Feature importance was evaluated by the Random Forest algorithm and was impurity-based (the sum over the number of splits—across all trees—that included the feature, proportionally to the number of samples split). A feature was selected for inclusion in the dosiomics signature if it was, on average, among the 30 most important features according to the Random Forest while having been selected by the Elastic Net. Features were ordered by feature class and then alphabetically.

### 2.6. Model Evaluation

The two possible types of wrong predictions have different implications: False Positives (or Type I error, i.e., falsely predicting that a survivor is at high risk of experiencing the event) would cost the CC survivors and the health system resources and time, while a False Negative (or Type II error, i.e., falsely predicting that a survivor is not at risk) could put CC survivors’ lives at risk. The statistical challenge is to accurately identify as many as possible high-risk individuals (True Positives) with the lowest possible ‘cost’ of wrong predictions: the so-called ‘avalanche problem’ [47]. Notably, Recall (sensitivity) is the metric that evaluates the algorithm’s ability to detect True Positives and not misclass them falsely as Negatives. On the other hand, Specificity is the probability of correctly identifying a survivor that will not experience the event; therefore, it evaluates the ‘cost’ of the algorithm in terms of False Positives. Thus, in this specific medical application, a low Recall means that the algorithm is inappropriate, while a low specificity is much more tolerable and secondary in terms of priorities for improvement. Finally, Balanced Accuracy is the average of sensitivity (Recall) and specificity (weighted Recall), and AUC is the area under the ROC curve (the integral of the curve of sensitivity against 1-specificity at various threshold settings). Therefore, both metrics simultaneously combine multiple quadrants of the confusion matrix (True Positives, False Positives, True Negatives, and False Negatives), providing an in-depth evaluation of models.

### 2.7. Cohort Partition Based on Heart Dose Heterogeneity

To explore and work out the imbalanced classification problem, we proposed a partition of the data based on the assumption that heart–dose heterogeneity might be an important factor for the occurrence of VHD. Two potential features measure heterogeneity: entropy and uniformity, negatively correlated. We chose uniformity, a normalized measure (taking values between 0 and 1). Uniformity is calculated as the sum of squares of each intensity value:

(1) $$\mathrm{Uniformity}=\sum_{i=1}^{N_{g}}p{\left(i\right)}^{2}$$

where, in Equation (1), $N_{g}$ is the number of non-zero bins of intensity level, equally spaced from 0 with a width defined in the binwidth parameter, $p\left(i\right)=\frac{P(i)}{N_{p}}$ is the normalized first-order histogram P(i), and $N_{p}$ is the total number of voxels. This measures the homogeneity of the radiation dose distribution. In this study, it was only computed for the doses absorbed from the heart. A high uniformity (close to 1) is interpreted either as homogeneity in the dose distribution or a smaller range of discrete intensity values [42].

We trained the wtRF and the BRF on three cohorts: (i) the entire cohort (7488 survivors, 81 of whom experienced a VHD) using dummy feature values for the patients that had not been treated with radiotherapy by setting to 0 the dose level absorbed by the heart voxels, (ii) the sub-population that had been exposed to non-homogeneous heart radiation (3556 survivors with Uniformity < 1, 61 of whom experienced a VHD), and finally (iii) the sub-population with very heterogeneous heart doses (1963 survivors with uniformity < 0.1, 57 of whom experienced a VHD).

Analyses were performed with Python 3.8.13. Data analysis was carried out with the libraries pandas [48], numPy [49], seaborn [50], and matplotlib [51]; dosiomics were extracted with the pyradiomics library [42]; and the pipelines for the modeling were built with Scikit-learn [52] and imbalanced-learn [53]. The threshold of significance was set to 0.05.

## 3. Results

### 3.1. Descriptive Analysis

In Table 1 and Table 2, we gathered information on the FCCSS and the sub-cohorts, defined according to the value of heart dose uniformity: no treatment with radiotherapy, uniformity = 1, uniformity inside the range [0.1, 1), and uniformity < 0.1.

From the 7488 5-year survivors of the FCCSS with complete data, 81 experienced a VHD (≈1%). A total of 63 of the survivors that experienced the event had been treated with radiotherapy, among whom, 2 had a heart–dose uniformity = 1, 4 had a uniformity between 0.1 and 1, and 57 had a uniformity < 0.1. The prevalence of VHD among survivors with uniformity < 0.1 is, thus, 2.9%. In the sub-population with uniformity = 1, the average mean, median, and maximum dose to the heart were all very low (0.2, 0.2, and 0.4 Gy, respectively), as well as each of their maximum values (0.25, 0.25, and 0.26 Gy respectively). On the contrary, among survivors with uniformity < 0.1, the average mean, median, and maximum dose to the heart increased by three orders of magnitude. In Table 2, we gathered information on the repartition of CC types in each cohort part. It is noteworthy that 84% of the survivors of Hodgkin lymphoma (394) had heart dose uniformity < 0.1. In addition, among survivors treated for renal tumors, 47% (531) had heart dose uniformity below 0.1, 9% between 1 and 0.1, and the rest (44%) were not treated with radiotherapy. Finally, 35% of survivors treated for the central nervous system and miscellaneous intracranial and intraspinal neoplasms (395) were among the 1963 survivors with heart dose uniformity < 0.1.

### 3.2. Dosiomics versus Mean Heart Dose

We first trained the models on the entire FCCSS (Table 3, rows 1–4). According to the BA and the AUC, models based on either the MHD or the dosiomics features performed similarly when trained with the wtRF algorithm (within the margin of error for the BA and the AUC). Most of the metrics’ comparisons were not statistically significant, neither with the wtRF nor with the BRF, when the models were trained on the entire population (both treated and not treated with radiotherapy). According to the BA, the AUC, and the Sensitivity, the MHD-based and the dosiomics-based models performed equally well in our cohort. Specificity was higher with the MHD-based wtRF (0.90 > 0.88, *p*-value = 0.001) and also with the dosiomics-based BRF (0.86 > 0.84, *p*-value = 0.044). In the case of both types of algorithms—wtRF and BRF—the MHD and the dosiomics-based algorithms seemed to perform similarly.

We then trained the same forests on the sub-population with non-homogeneous doses to the heart (3556 out of the 3902 survivors that had been treated with radiotherapy, based on the heart–dose uniformity being <1—Table 3, rows 5–8). All models seemed to improve (overall, metrics are higher for both types of Random Forests, wtRF or BRF, and both heart radiation measures, MHD or dosiomics features). With the wtRF, comparisons were not statistically significant. With the BRF, the dosiomics-based approach significantly outperformed the MHD (Table 3 row 8), based on three out of four metrics (BA: 0.79 > 0.74, *p*-value 0.004; AUC: 0.86 > 0.83, *p*-value = 0.046; and Specificity: 0.79 > 0.76, *p*-value = 0.001).

Finally, we attempted a stricter cut-off for the cohort partition and trained the models on the sub-population with heart–dose uniformity < 0.1 (Table 3, rows 9–12). The dosiomics-based model outperforms the MHD with both algorithms according to the Specificity (0.82 > 0.79, *p*-value = 0.001 with the wtRF and 0.77 > 0.73, *p*-value = 0.002 with the BRF).

Models trained on the sub-population of the FCCSS with heart–dose uniformity < 1 performed better than models trained on the sub-population with heart–dose uniformity < 0.1.

### 3.3. Models Adjusted on Clinical Variables

We also attempted to train the models adjusted on clinical variables. MHD and dosiomics-based models performed similarly well. Aggregated performance metrics for models trained on the entire FCCSS (Table A2—lines 1–4) and the sub-populations with heart–dose uniformity < 1 (Table A2—lines 5–8) and 0.1 (Table A2—lines 9–12) are included in Appendix A.

### 3.4. Sensitivity Analysis According to the Type of First Childhood Cancer

Table A3, in Appendix A, presents the results of a sensitivity analysis. We trained the models on survivors that had been treated for Hodgkin lymphoma, central nervous system malignancies, and renal tumors. Aggregated metrics and *p*-values are presented for non-adjusted and adjusted models. Comparison were not statistically significant and we cannot conclude that one model would outperform the others.

### 3.5. Dosiomics Signature

In Table 4, we provide information on the most important features by population (FCCSS, uniformity < 1, and uniformity < 0.1) and on whether they were selected as one of the most important features by each type of random forest (weighted and balanced). We present descriptives of the following 22 features that we propose as a dosiomics signature of a late VHD in the FCCSS:

- First order statistics: Tenth percentile, ninetieth percentile, energy, kurtosis, mean heart dose, median heart dose, minimum heart dose, root mean squared, total energy;
- GLCM: Autocorrelation, IDMN, IDN, joint average, sum average;
- GLDM: High gray level emphasis, large dependence high gray level emphasis, small dependence high gray level emphasis;
- GLRLM: High gray level run emphasis, long run high gray level emphasis, short run high gray level emphasis;
- GLSZM: Gigh gray level zone emphasis, small area high gray level emphasis.

**Figure 3:**
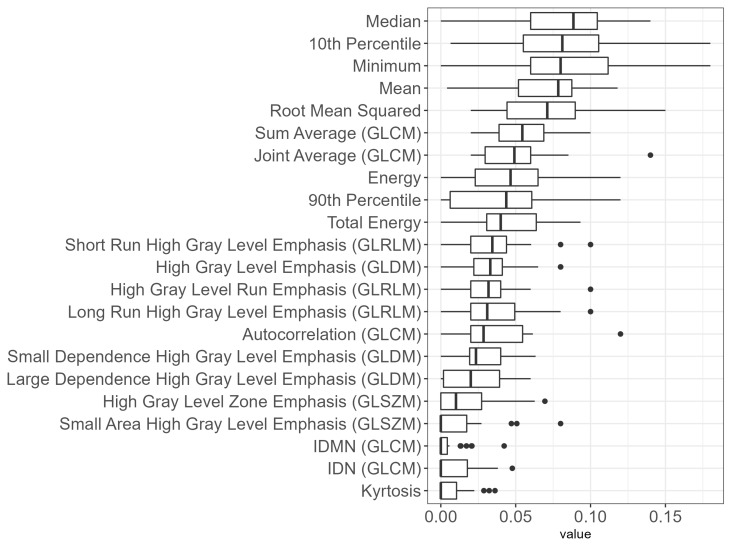
Boxplots of feature importance (aggregated over 30 train–test iterations) for the BRF trained on the population with uniformity < 1.

Additionally, boxplots describing variable importance in the BRF trained in the sub-population with uniformity < 1 are provided in Figure 3. We can observe that the median and the mean heart dose sort among the 5 most important features, along with the 10th dose percentile, the minimum, and the Root Mean Squared.

## 4. Discussion

The main finding of this study is that a random forest performs better in predicting CC survivors at risk of a radiation-induced VHD under a selection of dosiomics features describing the heart dose in comparison to the mean heart dose, and comparisons are statistically significant when applied to a population with some heterogeneity. We found a dosiomics signature of cardiac doses for the prediction of a late VHD in the FCCSS. To the best of our knowledge, this is the first study that explores the role of dosiomics features in the occurrence of a late VHD after treatment for a CC.

The particularity of the FCCSS is that it is the only study with a whole-body voxelized dosimetry reconstruction available for almost every participant that was treated with radiotherapy. This allows an in-depth investigation of the distribution of radiation dose and, in combination with the information on other treatments and interventions in the context of childhood cancer treatment, the long follow-up duration with available medical records, the access to the French Health Insurance Information System, as well as the adapted self-questionnaires may lead to reliable analyses that can be incorporated into international guidelines for rigorous and effective personalized follow-up with childhood cancer survivors.

Concerning the risk of VHD in particular, there is an established risk of VHD when strong doses are absorbed by heart tissues during treatment for adult [20,54] or childhood cancer [13,22,23], and there exist hypotheses on the role of low and moderate doses [16,18,23]. Meanwhile, studies claim that no level of radiation dose to the heart can be safe [55]. The aim of this study was to explore the effect of radiation doses absorbed by the heart by taking into account the heterogeneity of the dose. For that matter, we chose to extract dosiomics features from the dose matrices, a method that is becoming popular [56] and provides insight into the spatial and statistical characteristics of radiation dose.

### 4.1. The Role of Heterogeneity of the Heart Dose in Late Valvular Heart Disease

We proposed a sensitivity analysis, based on the heart dose uniformity. We observed that predictions improved when models were trained on the sub-population of the FCCSS with heart dose uniformity < 1, in comparison to models trained on the sub-population of the FCCSS with heart dose uniformity < 0.1. We hypothesize that the heart–dose heterogeneity is in fact a meaningful factor, in the sense that some of the features probably influence the predictions of survivors with heterogeneous doses. Therefore, the model was unable to distinguish survivors most at-risk to experience VHD when trained among survivors with a small uniformity range. This is one of the most fruitful results of this study.

We also included models trained on the entire FCCSS cohort, that contained survivors treated and not treated with radiotherapy. The model underperforms in comparison with the models trained on the sub-population of the FCCSS with heart dose uniformity < 1. Based on the assumption that cardiac radiation dose is not the only risk-factor responsible for a VHD, a dosiomics-based model is inappropriate for prediction for the non-irradiaded part of the cohort: the non-irradiated survivors that experience a VHD will always be incorrectly sorted in a model based on the radiation-induced risk.

Our main objective was to explore whether we can go beyond the use of the mean heart dose as an explanatory variable in the risk model. The idea was, thus, to see if descriptive statistics of the dose distribution, other than the mean dose, could carry additional information to improve predictions. When the dose distribution is uniform or nearly uniform, the mean dose is a sufficient descriptor of the distribution: other indicators might bring useful additional information only in the case of heterogeneous distributions. This part of the study aimed at investigating the effect of dose heterogeneity, not in itself, but as a criterion to discriminate cases where mean dose is likely to be a sufficient descriptor.

### 4.2. Model Choice and Performance

In [44], weighted and balanced random forests both improved prediction of the minority class in comparison to other algorithms. In our study, comparisons held between models with different predictors; comparing different types of algorithms was not one of the objectives in this study. Among performance metrics, Sensitivity (or Recall or True Positive Rate) is the most important for this application. It illustrates the existence of false negatives, whether all survivors who experienced the event were correctly sorted as high-risk. We also observed some models outperforming others based on Specificity. However, improving Specificity is a secondary objective of prediction models, as it evaluates the false positives. Therefore, between two models with contradictory results, we would choose the one with the highest True Positive Rate.

The two models with the highest Sensitivity are the MHD-based and the dosiomics-based BRF adjusted on clinical variables and trained on the sub-population with heart–dose uniformity < 1 (0.8 and 0.82 respectively—Table A2). However, the comparison between them is not statistically significant, and we cannot conclude if one of them outperforms the other. Next-highest is the dosiomics-based BRF, trained on the same population without adjustment on clinical variables (0.78—Table 3). In this scenario, the difference from the sensitivity of the MHD model (0.73) is close to being statistically significant. Taking into account that the other three metrics are significantly higher in comparison to the MHD-based model, we can derive that the dosiomics-based BRF trained on the sub-population with heart dose uniformity < 1 is the best-performing model in this study. Based on these observations, we conclude that the distribution of the radiation dose to the heart plays a complicated role in the occurrence of a VHD, which cannot be entirely captured by the MHD.

### 4.3. The Dosiomics Signature

The dosiomics signature can reflect the spatial complexity of the radiation dose and its association with the occurrence of a late VHD. It is noteworthy that, apart from very few exceptions, the two types of random forest evaluate the same variables as important on each sub-cohort. We observe that, in any case, MHD is among the most important features.

All of the features selected when models are trained among survivors with uniformity < 0.1 are also selected in at least one more model, trained on a larger population that includes survivors with higher heart dose uniformity (uniformity < 1 and the entire FCCSS). All models select energy and total energy, which depend on the magnitude of the voxel values, in the region of interest and, according to the authors [42], are volume-confounded. The mean and median heart dose as well as the root mean square, among the most important features of the model that seems to stand out (BRF on the sub-population with Uniformity < 1), are selected by all models.

GLCM features indicate how often pairs of voxels with specific values and in a specified spatial relationship occur. According to the authors, the sum average measures the relationship between pairs of voxels with lower intensity values and pairs of voxels with higher intensity values. We could, therefore, hypothesize that the sum average provides information on the effect of low doses in the occurrence of a late VHD. On the contrary, the high gray level emphasis and the small dependence high gray level emphasis from the GLDM class of features, as well as the GLRLM and GLSZM classes, cover different aspects of the effect of high dose levels in the prediction of a late VHD.

### 4.4. Limitations

One inconvenience of the method of this paper is that the interpretability of the dosiomics features is not always obvious, since most of the features are not widely used for statistical analyses. Also, dosiomics features are not directly extracted from the treatment-planning syste; it is, therefore, not always simple for the medical staff to incorporate them into prediction models.

Concerning the content of the data, a limitation also derives from the lack of information on comorbidities. Data related to comorbidities could improve prediction algorithms’ performance and the reliability of the results. Also, dose reconstruction comes with unavoidable uncertainties: a residual level of 2 to 5% in inaccuracy is generally observed for the dose at the organ of interest. The primary sources of uncertainty associated with dose estimation are (i) imaging of patient anatomy, (ii) reconstruction of the RT treatment plan, (iii) characterization of the irradiation source, and (iv) measurements or calculation of the dose distributions [39,40,57,58]. We assume that the voxelized dataset we are treating is sufficiently reliable. However, the advantage of this study is that the pipeline will still be applicable when uncertainties will have been removed from the dosimetric reconstruction.

The most important limitation is the lack of a validation set, a common problem in this type of study [59]. The number of events in the cohort is too low. Therefore, further partitioning the population to put aside a validation set would lead to loss of critical information necessary for the training. We decided the best strategy to eliminate some uncertainty from the results was to use the whole cohort in train–test partitioning and aggregate the results of 30 random stratified splits. External validation is, therefore, necessary. In this study, we aimed to propose a signal on the cardiac dosiomics signature for a late VHD, as well as a suggestion to incorporate information on the dose heterogeneity into the design of prediction algorithms and TPS guidelines.

### 4.5. Perspectives

For recently treated patients, data are automatically generated and can be archived [60], and for contoured organs of interest, the voxelized dose distribution can be extracted without significant cost. Therefore, these data can be used to derive dose–volume histograms, but also can be used as inputs for dosiomics analyses for radiation therapy side effects risk assessment.

Radiotherapists do their best to protect vital organs from strong radiation exposure [61]. However, it is still unclear if and how harmful exposure to low and moderate doses to the heart [62] could be. Meanwhile, while recent advancements make high MHD increasingly rare nowadays, novel radiotherapy delivery techniques such as IMRT or VMAT may increase the heart-volume receiving low-to-moderate radiation doses (<15 Gy). Dosiomics features could provide useful insight on the effect of spatially heterogeneous doses on the occurrence of late effects including VHD. Extracting dosiomics features directly from the treatment-planning system could be an interesting and useful perspective in this case.

For survivors who have received these treatments above a certain dose, several international guidelines recommend the completion of lifelong regular echocardiograms to allow earlier detection of asymptomatic cardiomyopathy, and thus reduce or delay sequelae by treating it. The recommended frequency of echocardiography ranges from every year to every five years, depending on the guidelines [63]. Even though the production of automated dosiomics applications is not yet a reality, risk models are already in use to design personalized follow-ups for each survivor.

Although still in its preliminary stage, our work paves the way toward an integrated optimization tool for recommending personalized follow-up protocols adapted to each patients’ health history [64]. In addition, the creation of a new branch of cardiology, “cardio-oncology”, with the aims of preventing cardiovascular complications related to antineoplastic treatment, achieving early diagnosis and treatment of any complications, and allowing completion of the expected antineoplastic treatment [65], should increase the offer of care for cancer survivors and encourage research in cardio-oncology. Defining the follow-up protocol is a delicate problem, with potentially dramatic consequences in case of maladjustment. The solution involving all sorts of screening exams at a high frequency would not be sustainable, both economically speaking and in terms of patients’ comfort and even safety. A perspective is, therefore, to turn to cost-effectiveness analysis.

## 5. Conclusions

Dosiomics are proving to be a promising strategy for exploring the radiation dose distribution and exposing information on the underlying pathophysiology of radiation-induced pathologies. The dosiomics-based BRF is the only model in this predictive attempt that, when compared to the relevant MHD-based model, stands out, and this comparison is statistically significant. This result could prove beneficial in identifying high-risk individuals even in a context where detailed clinical data are not available, but dosimetry data are available. If these findings hold, the dosiomics signature may be incorporated into machine learning classification algorithms for radiation-induced VHD risk assessment.

## Figures and Tables

**Table 1:** Descriptive table of the cohort (FCCSS) in the first column; then by radiotherapy status: survivors that had not been treated with radiotherapy (No RT), and survivors that had been treated with radiotherapy and had a heart dose uniformity = 1, between 0.1 and 1, and finally <0.1.

	FCCSS ^1^	No RT ^2^	Uniformity = 1	Uniformity in [0.1, 1)	Uniformity < 0.1
Total	7488	3586	346	1593	1963
VHD ^3^	81 (1.08%)	18 (0.5%)	2 (0.58%)	4 (0.25%)	57 (2.9%)
**Age at CC ^4^ diagnosis**	6.62 [0–20.61]	6.18 [0–20.41]	6.01 [0–18.41]	7.08 [0–20.28]	7.17 [020.61]
**Year at CC diagnosis**	1984 [1946–2000]	1988 [1949–2000]	1983 [1951–2000]	1982 [1946–2000]	1980 [1948–2000]
**Attained age**	37.76 [5.39–79.83]	35.79 [5.392–76.37]	39.37 [7.27–79.83]	38.94 [6.16–78.65]	40.12 [6.66–77.82]
**Biological Sex**					
Male	3384 (45.19%)	1622 (45.23%)	146 (42.2%)	701 (44.01%)	915 (46.61%)
Female	4104 (54.81%)	1964 (54.77%)	200 (57.8%)	892 (55.99%)	1048 (53.39%)
**Chemotherapy**					
No	1828 (24.41%)	957 (26.69%)	109 (31.5%)	480 (30.13%)	282 (14.37%)
Yes	5660 (75.59%)	2629 (73.31%)	237 (68.5%)	1113 (69.87%)	1681 (85.63%)
**Mean dose to the heart**	6.82 [0–61.20]	0 [0–0]	0.02 [0–0.25]	0.98 [0–37.65]	12.76 [0–61.20]
**Median dose to the heart**	6.75 [0–67.54]	0 [0–0]	0.02 [0–0.25]	0.88 [0–37.66]	12.69 [0–67.54]
**Maximum dose to the heart**	13.68 [0–109.43]	0 [0–0]	0.04 [0–0.26]	2.18 [0.1–60.28]	25.424 [1.326–109.43]
**Heart dose uniformity**	0.27 [0.003–1]	1 [1–1]	1 [1–1]	0.4 [0.1–1)	0.036 [0.003–0.1]

For continuous variables, the average is given as well as minimum and maximum (average [min–max]). For categorical variables, percentages are calculated over the total of the relevant sub-population. ^1^ French Childhood Survivors Study; ^2^ No Radiotherapy; ^3^ Valvular Heart Disease; ^4^ Childhood Cancer.

**Table 2:** The distribution of the type of first cancer in the cohort (FCCSS) in the first column; then by radiotherapy status: survivors that had been treated without radiotherapy (No RT), uniformity of radiation dose to the heart = 1, between 0.1 and 1, and <0.1.

	FCCSS ^1^	No RT ^2^	Uniformity = 1	Uniformity in [0.1, 1)	Uniformity < 0.1
Total	7488	3586 (48%)	346 (5%)	1593 (21%)	1963 (26%)
VHD ^3^	81	18 (22%)	2 (2%)	4 (5%)	57 (70%)
**Type of CC ^4^:**					
Hodgkin lymphoma	471	27 (6%)	5 (1%)	45 (10%)	394 (84%)
Other lymphomas and reticuloendothelial neoplasms	788	540 (69%)	16 (2%)	158 (20%)	74 (9%)
CNS and miscellaneous intracranial and intraspinal neoplasms	1124	160 (14%)	17 (2%)	552 (49%)	395 (35%)
Neuroblastoma and other peripheral nervous cell tumors	1028	646 (63%)	12 (1%)	144 (14%)	226 (22%)
Retinoblastoma	519	310 (60%)	114 (22%)	91 (18%)	4 (1%)
Renal tumors	1136	503 (44%)	0 (0%)	102 (9%)	531 (47%)
Hepatic tumors	79	62 (78%)	0 (0%)	5 (6%)	12 (15%)
Malignant bone tumors	679	392 (58%)	64 (9%)	124 (18%)	99 (15%)
Soft tissue and other extraosseous sarcomas	846	387 (46%)	99 (12%)	261 (31%)	99 (12%)
Germ cell tumors, trophoblastic tumors, and neoplasms of gonads	469	332 (71%)	6 (1%)	65 (14%)	66 (14%)
Other	349	227 (65%)	13 (4%)	46 (13%)	63 (18%)

Percentages are calculated over the cohort totals (column *FCCSS*). ^1^ French Childhood Survivors Study; ^2^ No Radiotherapy; ^3^ Valvular Heart Disease; ^4^ Childhood Cancer.

**Table 3:** Performance metrics, derived from training forests on the FCCSS and two sub-populations of the FCCSS (the part of the cohort with heart dose uniformity < 1 and the part of the cohort with heart dose uniformity < 0.1), according to two types of classification algorithms (weighted Random Forest—wtRF, and Balanced Random Forest—BRF), where the radiation-induced risk is explained by either the mean heart dose (MHD) or a selection of dosiomics features. Results are aggregated over the 30 instances of train–test spitting, and here we present the mean ± standard deviation of each metric.

		Heart Radiation Measure	Balanced Accuracy	AUC ROC	Sensitivity (Recall)	Specificity	
FCCSS	wtRF	**Mean** **heart dose**	0.74 ± 0.04	0.77 ± 0.051	0.57 ± 0.083	**0.90 ± 0.019**	
		**Dosiomics** **features**	0.74 ± 0.038	0.77 ± 0.047	0.59 ± 0.075	0.88 ± 0.015	
	*p*-values		*0.792*	*0.883*	*0.319*	*0.001*	
	BRF	**Mean** **heart dose**	0.73 ± 0.04	0.76 ± 0.046	0.61 ± 0.088	0.84 ± 0.034	
		**Dosiomics** **features**	0.74 ± 0.039	0.77 ± 0.051	0.62 ± 0.074	**0.86 ± 0.018**	4
	*p*-values		*0.234*	*0.358*	*0.627*	*0.044*	
Uniformity < 1	wtRF	**Mean** **heart dose**	0.78 ± 0.057	0.85 ± 0.059	0.72 ± 0.127	0.84 ± 0.029	
		**Dosiomics** **features**	0.78 ± 0.057	0.86 ± 0.059	0.73 ± 0.126	0.83 ± 0.031	
	*p*-values		*0.981*	*0.483*	*0.617*	*0.057*	
	BRF	**Mean** **heart dose**	0.74 ± 0.054	0.83 ± 0.057	0.73 ± 0.113	0.76 ± 0.043	
		**Dosiomics** **features**	**0.79 ± 0.056**	**0.86 ± 0.057**	0.78 ± 0.113	**0.79 ± 0.021**	8
	*p*-values		*0.004*	*0.046*	*0.08*	*<0.001*	
Uniformity < 0.1	wtRF	**Mean** **heart dose**	0.76 ± 0.068	0.81 ± 0.069	0.71 ± 0.146	0.79 ± 0.031	
		**Dosiomics** **features**	0.76 ± 0.062	0.82 ± 0.073	0.69 ± 0.13	**0.82 ± 0.026**	
	*p*-values		*0.909*	*0.773*	*0.4*	*0.001*	
	BRF	**Mean** **heart dose**	0.72 ± 0.076	0.79 ± 0.064	0.72 ± 0.151	0.73 ± 0.052	
		**Dosiomics** **features**	0.75 ± 0.056	0.8 ± 0.071	0.74 ± 0.126	**0.77 ± 0.028**	12
	*p*-values		*0.162*	*0.437*	*0.701*	*0.002*	

The last column corresponds to the enumeration of the table lines. *p*-values correspond to two-sided *t*-tests. The bolded metrics’ values are the ones that, compared to the model of the same type of forest but with a different heart radiation measure, are significantly higher.

**Table 4:** Dosiomics signature according to the sub-population (FCCSS, uniformity < 1 and uniformity < 0.1), and type of random forest (weighted or balanced).

	FCCSS			Uniformity < 1			Uniformity < 0.1		
Features	wtRF	BRF	Average [min–max]	wtRF	BRF	Average [min–max]	wtRF	BRF	Average [min–max]
**First Order Statistics: **									
10th percentile	✔	✔	1.78 [0–49.23]	✔	✔	3.75 [0–49.23]	✔	✔	6.18 [0–49.23]
90th percentile	✔	✔	5.37 [0–89.78]			11.31 [0–89.78]			19.36 [1.01–89.78]
energy	✔	✔	3.7 × 10^6^ [0–2.1 × 10^8^]	✔	✔	7.9 × 10^6^ [2.49–2.1 × 10^8^]	✔	✔	14 × 10^6^ [8.4 × 10^3^–2.1 × 10^8^]
kyrtosis		✔	3.49 [0–1753.9]			7.14 [1.1–1753.9]			6.03 [1.1–115.99]
mean heart dose	✔	✔	3.55 [0–61.09]	✔	✔	7.48 [0–61.09]	✔	✔	12.75 [0.64–61.09]
median heart dose	✔	✔	3.51 [0–67.91]	✔	✔	7.4 [0–67.91]	✔	✔	12.68 [0.44–67.91]
minimum heart dose	✔	✔	0.88 [0–38.24]	✔	✔	1.85 [0–38.24]			2.88 [0–38.24]
root mean squared	✔	✔	3.98 [0–64.33]	✔	✔	8.37 [0.01–64.33]	✔	✔	14.27 [0.7–64.33]
total energy	✔	✔	3 × 10^7^ [0–1.7 × 10^9^]	✔	✔	6.3 × 10^7^ [19.89–1.7 × 10^9^]	✔	✔	11 × 10^7^ [6.7 × 10^4^–1.7 × 10^9^]
**GLCM:**									
autocorrelation	✔	✔	0.58 × 10^4^ [1–3.1 × 10^5^]	✔	✔	1.2 × 10^4^ [1–3.1 × 10^5^]	✔	✔	2.1 × 10^4^ [41–3.1 × 10^5^]
IDMN		✔	1 [0.86–1]		✔	0.99 [0.86–1]			0.99 [0.86–1]
IDN		✔	0.99 [0.83–1]		✔	0.98 [0.83–1]	✔	✔	0.98 [0.83–1]
joint average	✔	✔	27.72 [1–512.79]	✔	✔	57.27 [1–512.79]	✔	✔	99.75 [5.38–512.79]
sum average	✔	✔	54.97 [1–10^4^]	✔	✔	114.54 [2–10^4^]	✔	✔	199.49 [10.76–10^4^]
**GLDM:**									
high gray level emphasis	✔	✔	0.59 × 10^4^ [1–3.1 × 10^5^]	✔	✔	1.2 × 10^4^ [1–3.1 × 10^5^]	✔	✔	2.2 × 10^4^ [42–3.1 × 10^5^]
large dependence high gray level emphasis	✔	✔	0.89 × 10^6^ [1–7.9 × 10^7^]			1.8 × 10^6^ [593–7.9 × 10^7^]			3.3 × 10^6^ [4.2 × 10^3^–7.9 × 10^7^]
small dependence high gray level emphasis	✔	✔	325.95 [0–39,643.4]	✔	✔	685.36 [0–39,643.4]	✔	✔	1239.17 [0.18–39,643.4]
**GLRLM:**									
high gray level run emphasis	✔	✔	6120.11 [1–321,807.62]	✔	✔	12,886.24 [1–321,807.62]	✔	✔	23,021.99 [45.97–321,807.62]
long run high gray level emphasis	✔	✔	55,488.09 [1–9,755,180.03]	✔	✔	116,805.48 [77.31–9,755,180.03]	✔	✔	205,185.69 [514.48–9,755,180.03]
short run high gray level emphasis	✔	✔	4118.5 [0.05–247,740.25]	✔	✔	8671.47 [0.07–247,740.25]	✔	✔	15,560.49 [14.08–247,740.25]
**GLSZM:**									
high gray level zone emphasis	✔	✔	6717.88 [1–347,651.5]			14,144.98 [1.2–347,651.5]			24,962.32 [50.85–347,651.5]
small area high gray level emphasis	✔	✔	1206.64 [0–99,793.65]			2539.85 [0–99,793.65]			4533 [0.09–99,793.65]

A check mark indicates that the feature was among the 30 most important of the model (averaged on 30 iterations). All of the features were selected via Elastic Net at least 25 out of 30 times.

## Data Availability

The datasets used and/or analyzed during the current study are available from the corresponding author upon reasonable request.

## Abbreviations

The following abbreviations are used in this manuscript:

VHD	Valvular Heart Disease
CC	Childhood Cancer
FCCSS	French Childhood Cancer Survivors Study
wtRF	weighted Random Forest
BRF	Balanced Random Forest
MHD	Mean Heart Dose

## Appendix A

**Table A1:** The full list of calculated features.

Feature Class	First-Order Statistics	Gray Level Co-Occurrence Matrix (GLCM)	Gray Level Run Length Matrix (GLRLM)	Gray Level Size Zone Matrix (GLSZM)	Gray Level Dependence Matrix (GLDM)	Neighbouring Gray Tone Difference Matrix (NGLDM)
**Number of features**	18	24	16	16	14	5
	mean heart dose (MHD)	autocorrelation	gray level non-uniformity	gray level non-uniformity	dependence entropy	busyness
	median	cluster prominence	non-uniformity normalized	gray level non-uniformity normalized	dependence non-uniformity	coarseness
	minimum	cluster shade	gray level variance	gray level variance	dependence non-uniformity normalized	complexity
	maximum	cluster tendency	high gray level run emphasis	high gray level zone emphasis	dependence variance	contrast
	variance	contrast	long run emphasis	large area emphasis	gray level non-uniformity	strength
	skewness	correlation	long run high gray level emphasis	large area high gray level emphasis	gray level variance	
	kurtosis	difference average	long run low gray level emphasis	large area low gray level emphasis	high gray level emphasis	
	entropy	difference entropy	low gray level run emphasis	low gray level zone emphasis	large dependence emphasis	
	uniformity	difference variance	run entropy	size zone non-uniformity	large dependence high gray level emphasis	
	10th percentile	Inverse Difference (ID)	run length non-uniformity	size zone non-uniformity normalized	large dependence low gray level emphasis	
	90th percentile	Inverse Difference Moment (IDM)	run length non-uniformity normalized	small area emphasis	low gray level emphasis	
	energy	Inverse Difference Moment Normalized (IDMN)	run percentage	small area high gray level emphasis	small dependence emphasis	
	total energy	Inverse Difference Normalized (IDN)	run variance	small area low gray level emphasis	small dependence high gray level emphasis	
	range	Informational Measure of Correlation 1 (IMC1)	short run emphasis	zone entropy	small dependence low gray level emphasis	
	interquartile range	Informational Measure of Correlation 2 (IMC2)	short run high gray level emphasis	zone 6 percentage		
	mean absolute deviation	inverse variance	short run low gray level emphasis	zone variance		
	robust mean absolute deviation	joint average				
	root mean squared	joint energy				
		joint entropy				
		Maximal Correlation Coefficient (MCC)				
		maximum probability				
		sum average				
		sum entropy				
		sum squares				

**Table A2:** Models trained and metrics calculated on the entire FCCSS (7488) cohort, and then on the sub-populations with heart–dose uniformity < 1 and <0.1, according to two types of classification algorithms (weighted Random Forest—wtRF, and Balanced Random Forest—BRF), where the radiation-induced risk is introduced by either the mean heart does—MHD, or a selection of dosiomics features. Results are aggregated over the 30 instances of train–test splitting, and here we present the mean ± standard deviation of each metric. Models in this table are adjusted on clinical variables: year and age of CC diagnosis, biological sex, and chemotherapy (y/n).

		Heart Radiation Measure	Balanced Accuracy	AUC ROC	Sensitivity (Recall)	Specificity	
FCCSS	wtRF	**Mean** **heart dose**	0.75 ± 0.041	**0.8** ± 0.044	0.62 ± 0.091	0.89 ± 0.027	
		**Dosiomics** **features**	0.74 ± 0.039	0.77 ± 0.051	0.6 ± 0.077	0.88 ± 0.012	
	*p*-values		*0.208*	*0.028*	*0.403*	*0.141*	
	BRF	**Mean** **heart dose**	0.76 ± 0.045	0.8 ± 0.054	0.68 ± 0.097	0.84 ± 0.029	
		**Dosiomics** **features**	0.74 ± 0.04	0.78 ± 0.054	0.65 ± 0.073	0.82 ± 0.023	4
	*p*-values		*0.057*	*0.126*	*0.169*	*0.092*	
Uniformity < 1	wtRF	**Mean** **heart dose**	0.81 ± 0.054	0.87 ± 0.048	0.74 ± 0.108	**0.87 ± 0.028**	
		**Dosiomics** **features**	0.78 ± 0.063	0.86 ± 0.057	0.73 ± 0.134	0.83 ± 0.028	
	*p*-value		*0.117*	*0.594*	*0.666*	*<0.001*	
	BRF	**Mean** **heart dose**	0.82 ± 0.053	0.88 ± 0.046	0.82 ± 0.106	**0.82 ± 0.023**	
		**Dosiomics** **features**	0.8 ± 0.062	0.86 ± 0.057	0.8 ± 0.123	0.8 ± 0.019	8
	*p*-values		*0.171*	*0.219*	*0.526*	*<0.001*	
Uniformity <0.1	wtRF	**Mean** **heart dose**	0.76 ± 0.077	0.85 ± 0.052	0.69 ± 0.155	0.83 ± 0.025	
		**Dosiomics** **features**	0.77 ± 0.061	0.85 ± 0.057	0.71 ± 0.145	0.83 ± 0.031	
	*p*-values		*0.718*	*0.086*	*0.811*	*0.482*	
	BRF	**Mean** **heart dose**	0.77 ± 0.059	0.84 ± 0.057	0.76 ± 0.123	0.78 ± 0.026	
		**Dosiomics** **features**	0.78 ± 0.049	0.86 ± 0.05	0.76 ± 0.113	0.8 ± 0.032	12
	*p*-values		*0.779*	*0.183*	*0.673*	*0.482*	

The last column corresponds to the enumeration of the table lines. *p*-values correspond to two-sided *t*-tests. The bolded metrics’ values are the ones that, when compared to the model of the same type of forest but with a different heart radiation measure, are significantly higher.

**Table A3:** Comparison of the metrics of 4 models: MHD, dosiomics features and their adjusted versions in Hodgkin lymphoma, central nervous system malignancies, and renal tumor survivors.

		Heart Radiation Measure	Balanced Accuracy	AUC ROC	Sensitivity (Recall)	Specificity	
Non Adjusted models	wtRF	**Mean** **heart dose**	0.78 ± 0.09	0.82 ± 0.071	0.7 ± 0.199	0.86 ± 0.033	
		**Dosiomics** **features**	0.75 ± 0.08	0.83 ± 0.053	0.66 ± 0.182	0.85 ± 0.028	
	*p*-values		*0.527*	*0.751*	*0.628*	*0.588*	
	BRF	**Mean** **heart dose**	0.78 ± 0.086	0.83 ± 0.062	0.73 ± 0.2	0.82 ± 0.035	
		**Dosiomics** **features**	0.76 ± 0.072	0.83 ± 0.065	0.71 ± 0.166	0.81 ± 0.029	4
	*p*-values		*0.712*	*0.870*	*0.801*	*0.705*	
Adjusted models	wtRF	**Mean** **heart dose**	0.79 ± 0.086	0.87 ± 0.059	0.71 ± 0.187	0.87 ± 0.033	
		**Dosiomics** **features**	0.76 ± 0.088	0.83 ± 0.059	0.67 ± 0.192	0.84 ± 0.028	
	*p*-values		*0.406*	*0.155*	*0.627*	*0.088*	
	BRF	**Mean** **heart dose**	0.8 ± 0.056	0.87 ± 0.059	0.76 ± 0.12	**0.84 ± 0.016**	
		**Dosiomics** **features**	0.78 ± 0.062	0.85 ± 0.064	0.73 ± 0.142	0.82 ± 0.027	8
	*p*-values		*0.394*	*0.544*	*0.723*	*0.022*	

The last column corresponds to the enumeration of the table lines. *p*-values correspond to two-sided *t*-tests. The bolded metrics’ values are the ones that, compared to the model of the same type of forest but with a different heart radiation measure, are significantly higher.
